# Effect of oral health promotion interventions on pregnant women dental caries: a field trial

**DOI:** 10.1186/s12903-022-02292-1

**Published:** 2022-07-08

**Authors:** Marzie Deghatipour, Zahra Ghorbani, Amir Hossein Mokhlesi, Shahla Ghanbari, Mahshid Namdari

**Affiliations:** 1 Present Address: Department of Community Oral Health, Dental School, Zahedan University of Medical Sciences, Zahedan, Iran; 2 Department of Community Oral Health, Dental School, Shahid Beheshti University of Medical Sciences, Tehran, Iran; 3 Present Address: Research Institute of Dental Sciences, Dental School, Shahid Beheshti University of Medical Sciences, Tehran, Iran; 4 Deputy for Health Affairs, Shahid Beheshti University of Medical Sciences, Tehran, Iran; 5 Department of Biostatistics, Faculty of Paramedical Sciences, Shahid Beheshti University of Medical Sciences, Tehran, Iran; 6 Oral and Dental Disease Research Center, Zahedan University of Medical Sciences, Zahedan, Iran

**Keywords:** Dental caries, Pregnant women, Oral health, Community-based interventions

## Abstract

**Background:**

Dental caries is a costly and very common disease, especially in pregnant women. Reasons such as not paying attention to oral health, poor diet and also lack of adequate education in this regard cause this to happen. Performing well-designed educational interventions using primary health system’s forces, can improve oral health of pregnant women and help control this disease. We conducted this study to evaluate the effectiveness of some oral health interventions on pregnant women dental caries.

**Methods:**

A field trial study was done in comprehensive Health Centers in Varamin, Tehran, Iran to assess 439 mothers’ dental health status from pregnancy up to 2 years after delivery in intervention (n = 239) and control groups (n = 200). Mothers in intervention groups received oral health-related education consisted of nutritional and behavioral messages via either of four methods: A: comprehensive method including all following methods together (n = 74), B: group discussion by dentists (n = 59), C: face to face education by primary health care providers (n = 53), and D: social network applications (n = 53); while those in control group only received routine maternal and oral health care. We used a questionnaire to collect mothers’ demographic, socioeconomic and dental care behavior data and also performed oral examinations to assess their DMFT at baseline and 24 months after delivery to evaluate the effectiveness of these educational oral health interventions.

**Results:**

From 454 mothers participated the examination session, 18 pregnant women discontinued during the follow-ups and 439 were remained with mean age of 27.47. In the intervention group, the frequency of daily brushing among women increased from 64% at baseline to 85.6% at the last follow-up and the mean D significantly decreased nearly 1unit at same period (P < 0.05). Most and least dental caries changes were in comprehensive intervention group and social network intervention group compared to other intervention groups, respectively.

**Conclusions:**

Performing educational interventions during and after pregnancy using various message delivery methods and messengers (oral health professionals and trained PHCPs), could improve oral health status and behaviors of pregnant and lactating mothers in a feasible and applicable manner.

## Background

Dental caries is the most prevalent global infectious diseases with considerable economic and quality-of-life burdens. There is enough evidence to conclude that poor oral health behaviors and bad dietary habits such as excessive consumption of sweets are important risk factors associated with dental caries [1, 2]. One of the groups most prone to tooth decay are pregnant women. According to reports, tooth decay in these people was up to 2.9 times more than normal people. They were also more likely to develop gingivitis and generally, their oral health was more at risk [3]. Also, periodontitis is a common condition in pregnancy, and these two conditions are related to each other due to various factors. Periodontitis during this period can lead to negative pregnancy outcomes such as preterm birth and low birth weight [4]. Maternal oral health status also can affect developing early childhood caries that may result in so many consequences for child’s health in the future [5, 6]. The importance of oral health before, during and after pregnancy has attracted the attention of policymakers, scientific foundations, agencies, and Primary Health Care Providers (PHCPs) who serve pregnant women and young children. It is recommended that pregnant women receive oral health education and self-care behavior education to prevent dental infections during pregnancy period [7]. A recent systematic review of oral health knowledge and awareness in pregnant women concluded that they had poor level of knowledge and awareness [8]. Also, another study on pregnant women reported that the majority of participants had received no instructions on oral health care during their pregnancy [9]. All of this evidence points to the importance of oral health education in pregnancy and the need to start planning for it.

Health promotion is defined as the “process of enabling people to increase control over and to improve their health. It moves beyond a focus on individual behavior toward a wide range of social and environmental interventions”. Health promotion goal can be to increase health‐related knowledge or awareness; making changes in different behavioral determinants such as beliefs, attitudes and habits and finally removing environmental and social barriers that influence health related behaviors [10]. Oral health promotion is crucial during pregnancy period and should contain educational programs and oral health care providing [11]. Majority of evidences underlined paying more attention to oral health education programs and interventions such as face to face and focus group discussion during pregnancy [6, 7, 12].

A recent review study revealed that a small number of oral health interventions for pregnant women covered the various dimensions needed to successfully promote oral health. Therefore, more evidence-based interventions with appropriate design are needed to teach oral health guidelines and achieve oral health promotion during pregnancy [12]. One of the things that can make oral health interventions better is integrating it with general health care in forms such as primary health care, according to new FDI World Dental Federation’s recommendation [13]. In the Islamic Republic of Iran reportedly, in 1995, oral health was integrated into Primary Health Care (PHC) to provide preventive intervention to the target group of mothers and children under 5 in all regions of Iran [14]. Free maternal and child health services were provided in each town’s Public Healthcare Centers, including pregnancy visits, baby growth monitoring and vaccination by PHCPs. The national population coverage of child care (vaccination and growth monitoring of newborns) and maternal care (antenatal care coverage) was reported 98% [15] and 94.3% [16] in public health services in Iran, respectively. PHC workers (PHCPs) were assigned to conduct educational and preventive interventions on mothers and children’s oral health as well as dentists to do dental treatments in the context of PHC setting in public health sector. However, a recent study in Iran revealed that the assigned preventive oral health care was not performed well enough and this integration did not result in improvements in oral health in the targeted population. The mean DMFT index for pregnant women in this study was 10.3, of which about 70% was D, which is not satisfactory and showed a serious need for treatment. Also, daily toothbrushing habit was less common compared to some other countries that indicates more educational efforts is needed [17].

Because dental caries is a multi-factorial disease, thus multiple interventions benefiting from different methods and settings are more effective than a single intervention. It is suggested that interventions should involve dietary advice such as lower sugar intake and increased consumption of fruits and vegetables, along with oral hygiene instructions, according to WHO’s recommendations [7].

According to results reported worldwide and especially in this country, oral health promotion intervention was planned and providers were assigned, but there was a gap which has led to ineffective efforts. Therefore, it is necessary to conduct a study and assess the efficacy of a feasible, available and not expensive interventions for pregnant and lactating women. The aim of this study was to evaluate the effect of oral health interventions on pregnant women dental caries as an indicator for oral health promotion outcome to achieve an effective intervention plan to help policymakers improve the oral health of mothers and children.

## Methods

### Study design and setting

A field trial was designed to evaluate the effect of oral health promotion interventions on mothers’ dental caries. This study was done in Pishva and Pakdasht. These cities are neighbors in Varamin region, Tehran province, Iran, and both are partially deprived and similar in socioeconomic status. Health centers for various interventions were selected in consultation with the local managers of Pishva region, considering the possibility of implementing the interventions.

### Sample size and selection

According to the previous study, the SD of decayed teeth of Iranian pregnant women was 4.40 [18]. Considering that the intervention is expected to lead to an 20% reduction in D (α = 0.05 with 80% of power), approximately, 200 subjects were needed to participate in each case and control group in this study. This article was part of a larger oral health project about maternal and child oral health promotion.

The target population comprised pregnant women (15 years and older) in the second/third trimester of pregnancy residing in Pishva and Pakdasht, seeking maternal care from the 18 Public Healthcare Centers. The research participants were recruited and followed up for 24 months after delivery. Recruitment began in July 2016 and the follow up procedure ended in November 2018. All registered pregnant women, in second/third trimester of pregnancy, with a pregnancy record (routine maternal care records) in Pishva and Pakdasht health centers were recalled by the Primary Health Care Providers (PHCPs) up to three times calls. Also, pregnant women with known systemic disease, high-risk pregnancy, long-term use of medications and those who did not respond to three times calls were excluded. From the 647 registered women, 454 attended the examination session. After the assessment of the inclusion and exclusion criteria, research team provided information about the study aims and intentions to the recruited women and then obtained informed consent from them. Verbal consent was taken from illiterate mothers. Participants were assigned to study groups in consultation with the local managers of the comprehensive health centers and based on their recommendations, as well as according to the population of each center and their distance to prevent contamination of the samples.

### Intervention

All the interventions were led by a dental public health Associate Professor and a trained dental public health PhD candidate in collaboration with the Health Deputy of Shahid Beheshti University of Medical Sciences. Interventions were consisted of offering nutritional and behavioral oral health-related messages and trainings, some tips from National Maternal and Child Oral Health Policy Center recommendations [7] and WHO Guideline on Sugars Intake for Adults and Children [19]. The educational content has been approved by the Department of Oral Health of Shahid Beheshti University of Medical Sciences. Nutritional messages consisted of mother and child healthy diet, encouraging daily intake of fruits and vegetables, restricting frequency of sweet consumption (e.g. juices, honey, chocolate, cookies, and sugar) in baby bottle.

Behavioral messages included teaching the correct method of tooth brushing, encouraging mothers to brush their teeth twice a day, using fluoridate tooth paste, clarifying the importance of dental visits during pregnancy and lactating period for both mother and newborn, using chewing xylitol gum, avoiding any use of tobacco products, using of finger tooth brush for child, avoiding breastfeeding during the night and sweet bottle feeding at all times. Interventions were performed in all eight Public Healthcare Centers in Pishva as the intervention group using the 4 following methods:

A Comprehensive intervention:
  This type of intervention was performed on 74 mothers at two health care centers and consisted of all the three mentioned interventions below.
B Performing group discussion sessions by dentists:
  Totally seven behavioral and nutritional educational sessions were performed every 3 months, from late pregnancy up to 18 months after delivery. Nearly 59 mothers participated in group sessions. Group sessions were held at the conference hall of healthcare centers and they were facilitated by a trained dentist, a nutritionist and a midwife in each center. Lectures were delivered by the dentist to provide advices on the educational tips mentioned above, group discussion (question and answer) about mothers and their newborn oral health behavioral and dietary issues. Main topics from previous sessions were asked and repeated in the next group session. At the first educational intervention, participants were given a pamphlet consisting of the given advices at the session, and a tube of fluoridated toothpaste accompanied with a tooth brush.
C Face to face advice by PHCPs at routine maternal and child care visits:
  All the PHCPs working in Pishva healthcare centers and health posts were trained during three workshops, focusing on the importance of pregnant women’s oral health and the essential role of PHCPs on improving mother and newborn’s oral health. Approximately 53 mothers were educated by these trained PHCPs. These sessions were implemented at pregnancy, 1, 6, 12 and 18 months after delivery, when mothers attended health care centers for routine baby checkups (vaccination and growth supervision of newborns). PHCPs educated mothers on nutritional and behavioral issues using posters, pamphlets, dentate manikin head model and tooth brush, and finger brush.
D Social networking applications:
  A Dental Public Health PhD candidate created a channel on Telegram social network application and sent 84 behavioral (n = 41) and nutritional (n = 43) contents consisted of audios, videos, and text messages to 53 mothers enrolled in healthcare centers. Participants received messages every week starting from pregnancy period until 18 months after the delivery.
  All 200 mothers in ten Public Healthcare Centers in Pakdasht received routine maternal and child health services (oral and general) as the control group.

### Data collection and variables

For data collection, oral examinations were done and a structured questionnaire was used. Oral examinations were performed at baseline (pregnancy period), 6 and 24 months after their delivery. Two trained and calibrated dentists (Kappa = 0.85) recorded Decayed (D), Missing (M), and Filled (F) teeth of each participant using cotton rolls, battery-operated lights and mouth mirror at the maternal care room in public health centers, according to the World Health Organization (WHO) oral health surveys basic methods [20]. Dentists were blinded to group allocation during oral examinations. At last, participants were informed of the results of their oral examinations and, if needed, were advised to visit a public dental service for treatments.

Questionnaire was filled by a Dental Public Health PhD candidate via face-to-face interviews with mothers at intervals similar to oral examinations. This questionnaire was used in research team’s previous study on mothers oral health status [17] and included demographic questions such as mother’s age, educational background, family size, and income, and some oral health behavior questions including daily frequency of tooth brushing, flossing and sweets consumption habits and also asking if they had a dental visit in the last 6 months. After completing oral examinations and interviews, all the study participants were given a tube of fluoridated tooth paste and a tooth brush. The correct brushing method was demonstrated to all of the participants and they were advised to brush at least twice a day.

Main outcome variables were D, M, and F at 24 months after delivery. Main explanatory variable was intervention group vs control group. Covariates including age, socio-economic status (SES) variables (income, education, family size) and potential mediators including behavioral variables (brushing, flossing, dental visit and sweet consumption) in the 6th month after delivery were considered were considered. All the analyses were controlled for baseline D, M, and F to consider the participants’ baseline oral health status.

### Statistical analysis

Statistical analysis and data preparation were done via IBM SPSS Statistics (version 19) software and STATA (version 14). For bivariate statistical analysis, Chi-square, Kruskal–Wallis, and Mann–Whitney U tests were used. Also, for modeling D and M, General Linear Model (Link function) was used, while due to the multiplicity of zero values in F, Poisson regression with zero inflation was used to model it. Adjusted models were used to study the association between explanatory variables and outcome variables. P-value less than 0.05 was considered statistically significant.

### Ethical issues

This study was approved by the Committee of Ethics in Research Affairs of Dental School, Shahid Beheshti University of Medical sciences (Code: IR.SBMU.DRC.REC.1397.003). Informed consent was taken from the mothers after providing information on study objectives and the confidentiality of the participant’s information throughout the study.

## Results

**Fig. 1:**
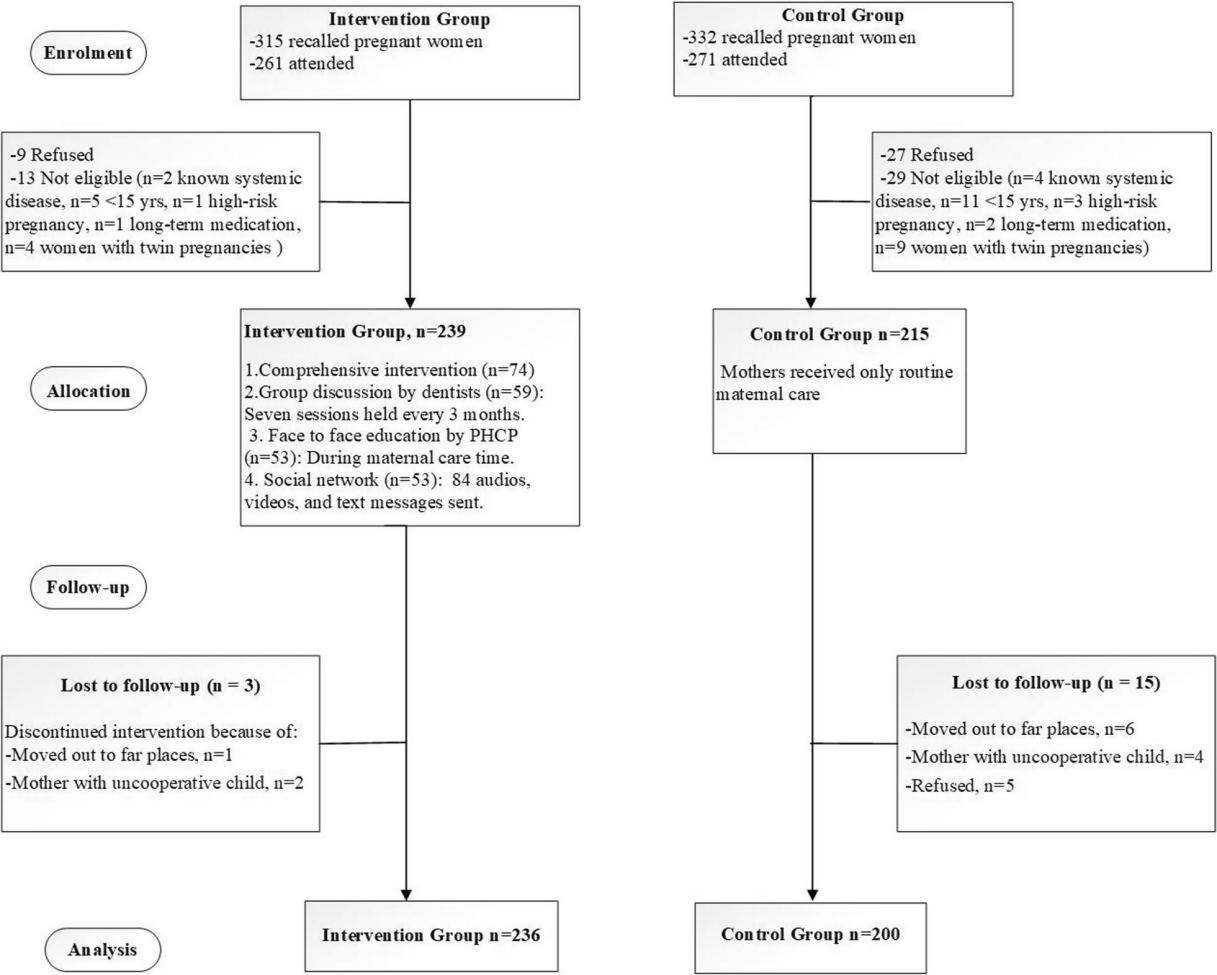
Flowchart of mother intervention study design

Amidst all 647 registered women, 454 (intervention, n = 239 and control, n = 215) participated the examination session. The flowchart of sampling is provided in Fig. 1. During the follow-up, totally, 18 pregnant women (intervention, n = 3 and control, n = 15) discontinued this study.

The mean (SD) age of the women in the intervention and control group was 27.05 (5.43) and 27.98 (5.76) respectively. About half of the mothers participated in the study were educated for a period of less than 12 years. Majority of mothers in both intervention and control group were located in low- and middle-income groups (Table 1).

**Table 1:** Participants distribution according to demographic, socioeconomic, dental care behaviors, and mother’s dental caries variables in intervention and control groups (n = 436)

Characteristics	Intervention (n = 236)				Control (n = 200)
	Comprehensive (n = 74)	Group discussion (n = 58)	Face to face by PHCPs (n = 52)	Social network (n = 52)	Control (n = 200)
	N (%)	N (%)	N (%)	N (%)	N (%)
Demographic variables					
Maternal age group					
15–25	22 (29.70)	21 (36.20)	16 (30.80)	19 (36.50)	58 (29.00)
25–35	45 (60.80)	29 (50.00)	31 (59.60)	27 (51.90)	112 (56.00)
35–44	7 (9.50)	8 (13.80)	5 (9.60)	6 (11.50)	30 (15.00)
Socioeconomic variables					
Maternal level of education					
Less than 12 years	42 (56.80)	28 (48.30)	31 (59.60)	28 (53.80)	116 (58.00)
12 years	27 (36.50)	28 (48.30)	18 (34.60)	18 (34.60)	71 (35.50)
More than 12 years	5 (6.80)	2 (3.40)	3 (5.80)	6 (11.50)	13 (6.50)
Family income					
Low	33 (44.60)	15 (25.90)	20 (38.50)	15 (28.80)	70 (35.00)
Middle	26 (35.10)	27 (46.60)	22 (42.30)	19 (36.50)	95 (47.50)
High	15 (20.30)	16 (27.60)	10 (19.20)	18 (34.60)	35 (17.50)
Family size					
3–4	50 (67.60)	40 (69)	36 (69.20)	40 (76.90)	132 (66.00)
5–6	24 (32.40)	18 (31)	16 (30.80)	12 (23.10)	68 (34.00)
Total	74 (100%)	58 (100%)	52 (100%)	52 (100%)	200 (100%)

At the last follow-up (24 months after delivery), participants in low-income group had more D compared to middle and high-income group. Also, more filled teeth were observed in participants who had educated for more than 12 years or visited a dentist in 6 months ago compared to others. Moreover, women in higher age group had more D, M, and F (P < 0.05, P < 0.001). More missing teeth also were explored in mothers with higher family size (P < 0.001) (Table 2).

**Table 2:** Demographic, socioeconomic and oral health of women at 24 months’ follow-up (n = 436)

Variables		N (%)	D Mean (SD)	M Mean (SD)	F Mean (SD)
*Demographic variables*					
Age group	15–25	169 (38.8)	6.64 (4.23)	1.51 (1.92)	1.17 (1.79)
	25–35	221(50.7)	6.40 (3.97)	2.57 (2.61)	1.80 (2.44)
	35–44	46 (10.6)	4.96 (2.89)	4.46(3.51)	2.85 (2.89)
	*P-value*		**0.05**^**a**^	** < 0.001**^**a**^	** < 0.001**^**a**^
Place	Intervention	236 (54.1)	6.72 (4.02)	2.53 (2.8)	1.78 (2.18)
	Control	200 (45.9)	5.89 (3.93)	2.16 (2.41)	1.54 (2.46)
	*P-value*		**0.01**^**b**^	0.16^b^	**0.03**^**b**^
*Indicators of socioeconomic status*					
Income	Low	152 (34.9)	7.98 (4.18)	2.38 (2.55)	1.55 (2.05)
	Middle	189 (43.3)	6.0 (3.17)	2.53 (2.54)	1.61 (2.06)
	High	95 (21.8)	4.4 (4.16)	1.99 (2.91)	1.98 (3.07)
	*P-value*		** < 0.001**^**a**^	**0.02**^**a**^	0.85^a^
Education (years of schooling)	Less than 12 years	187 (42.9)	6.68(4.21)	3.36 (4.33)	1.96 (2.49)
	12 years	224 (51.4)	6.76 (4.22)	2.21 (2.35)	1.20 (1.84)
	More than12 years	25 (5.7)	5.79 (3.63)	2.40 (2.64)	2.19 (2.67)
	*P-value*		0.09^a^	0.69^a^	** < 0.001**^**a**^
Family size	3–4	298(68.3)	6.23(3.98)	2.03(2.49)	1.68(2.39)
	5–6	138(31.7)	6.58(4.03)	3.06(2.79)	1.64(2.16)
	*P-value*	0.74^b^	0.46^b^	** < 0.001**^**b**^	0.843^b^
*Dental care behaviors*					
Brushing Habit (6 month)	Once a day or more	325 (74.50)	6.42 (4.03)	2.39 (2.47)	1.69 (2.22)
	Less than once a day	111 (25.50)	6.09 (3.91)	2.28 (3.07)	1.59 (2.58)
	*P-value*		0.40^b^	0.13^b^	0.23^b^
Flossing Habit (6 month)	once a day or more	98 (22.5)	6.51(3.84)	2.02 (2.23)	1.61 (2.33)
	Less than once a day	338 (77.5)	6.29 (4.04)	2.46 (2.73)	1.69 (2.32)
	*P-value*		0.43^b^	0.11^b^	0.56^b^
Dental Visit (6 month)	Yes	118 (27.1)	7.16 (4.02)	3.20 (2.84)	2.29 (1.97)
	No	318 (72.9)	6.04 (3.95)	2.05 (2.48)	1.44 (2.39)
	*P-value*		**0.01**^**b**^	** < 0.001**^**b**^	** < 0.001**^**b**^
Sweet Consumption	once a day	301 (69)	6.12 (3.89)	2.31 (2.57)	1.86 (2.57)
	more than once a day	135 (31)	6.84 (4.19)	2.47 (2.77)	1.24 (1.52)
	*P-value*		0.17^b^	0.55^b^	0.21^b^
Total	436(100%)	6.34 (3.99)	2.36 (2.63)	1.67 (2.32)	

Bold numbers: relationship significant at 5% level

^a^Krusal–Wallis test

^b^Mann–Whitney U-test

The frequency of daily brushing among women in intervention group increased from 64% at baseline to 85.6% at the last follow-up, while no change was observed in the control group. At baseline, one third of women in intervention group consumed sweet stuff more than once a day which decreased to 17% at the last follow-up. Also, having a dental visit in the previous year decreased from 58% to 1.5% in the control group whereas it increased from 43% to nearly 50% in the intervention group during the study period.

**Fig. 2:**
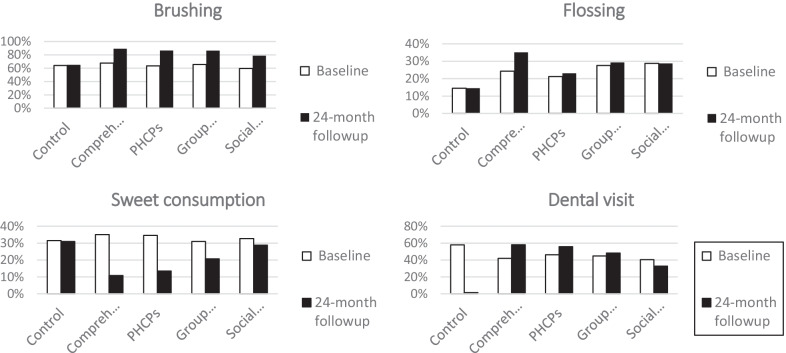
Brushing habit (brushing once a day or more), Flossing habit (flossing once a day or more), Sweet consumption habit (consume sweet more than once a day or more), and Dental visit (have any dental visits in the previous 12 months) in four intervention and control groups at baseline, and 24 months follow-up (by percentage %)

Women in intervention group especially those who underwent comprehensive intervention had more daily tooth brushing, flossing and less sweet consumption habits and also, having a dental visit has increased among them in the last 12 months at the last follow-up compared to the base line. Detailed information regarding brushing, and flossing, sweet consumption and dental visit were indicated in Fig. 2. Most and least dental caries changes were in comprehensive intervention group and social network intervention group compared to other intervention groups respectively. The mean D in intervention group significantly decreased from 7.60 at baseline to 6.72 at follow-up in 24 months after delivery (P < 0.05). Also, while the mean F in control group was more than intervention group at baseline, this amount surpassed in intervention group at the last follow-up.

**Fig. 3:**
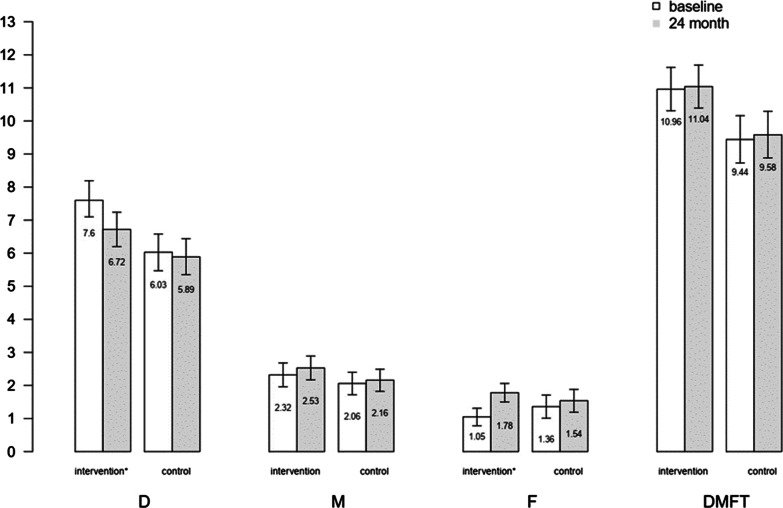
Mean and 95%CI for mean of D, M, F, and DMFT indicators in intervention field and control group at baseline and at 24 months follow-up

As shown in Fig. 3, either at baseline or at 24 months follow-up, the mean D and DMFT were higher in intervention group compared to control group. The mean D in intervention group significantly decreased from 7.60 at baseline to 6.72 at 24 months follow-up (P < 0.05). At baseline, the mean F in control group was more than intervention group, but at 24 months follow-up, this amount surpassed in intervention group.

The results of the last follow-up in Table 3 showed that the intervention group had 10% less decayed teeth compared to the control group after controlling for place, age, and SES (models 1–3). But in model 4 when oral health behavioral factors were controlled, no significant difference was detected. The number of missing teeth were not statistically different in intervention group, neither in adjusted models nor in unadjusted ones (Table 4).

**Table 3:** Association between demographic and dental care behaviors with number of decayed teeth at 24 months follow-up (n = 436)

D-24 months		Model1 P-value and CR (95% CI)	Model2 P-value and CR (95% CI)	Model3 P-value and CR (95% CI)	Model4 P-value and CR(95% CI)
Baseline D at pregnancy		< 0.001*	< 0.001*	< 0.001*	< 0.001*
Place	Intervention	< 0.02* and 0.92 (0.85, 0.99)	< 0.02* and 0.92 (0.85, 0.99)	< 0.03* and 0.92 (0.86, 0.99)	0.43 and 0.97 (0.89, 1.05)
Age			0.76	0.62	0.70
Family size				0.96	0.84
Education				0.50	0.69
Income	Low			0.02* and 1.15 (1.02, 1.29)	0.06 and 1.12 (0.99, 1.26)
	Middle			< 0.001* and 1.22 (1.09, 1.37)	< 0.001* and 1.22 (1.08, 1.36)
Daily brush (at 6 m)	Less than once				0.55 and 1.03 (0.94, 1.13)
Daily floss (at6m)	Less than once				0.82 and 0.99 (0.90, 1.08)
Sweet consumption (at 6 m)	Once and less				0.84 and 0.99 (0.91, 1.08)
Having Visited a dentist in the past 12 months (at 6 m)	Yes				< 0.001* and 1.27 (1.17, 1.39)

Bold numbers: relationship significant at 5% level

**Table 4:** Association between demographic and dental care behaviors with number of missing teeth at 24 months follow up (n = 436)

M-24 months		Model1 P-value and CR (95% CI)	Model2 P-value and CR (95% CI)	Model3P-value and CR (95% CI)	Model4 P-value and CR (95% CI)
Baseline M at pregnancy		< 0.001*	< 0.001*	< 0.001*	< 0.001*
Place	Intervention	0.76 and 1.01 (0.89, 1.15)	0.50 and 1.04 (0.92, 1.18)	0.56 and 1.03 (0.91, 1.18)	0.82 and 0.98 (0.85, 1.12)
Age			< 0.001*	0.52	0.35
Family size				0.004*	0.01*
Education				0.03*	0.09
Income	Low			< 0.001* and 1.39 (1.16, 1.18)	< 0.001* and 1.35 (1.12, 1.63)
	Middle			< 0.001* and 1.36 (1.14, 1.61)	0.002* and 1.32 (1.10, 1.57)
Daily brush (at 6 m)	Less than once				0.23 and 0.91 (0.78, 1.06)
Daily floss (at6m)	Less than once				0.56 and 1.04 (0.89, 1.23)
Having Visited a dentist in the past 12 months (at 6 m)	Yes				0.002* and 1.24 (1.08, 1.42)

GLM model with poisson link.function

Bold numbers: relationship significant at 5% level

Model 1: adjusted for baseline M and place

Model 2: adjusted for baseline M and place, and age

Model 3: adjusted for baseline M, place, age, education, and income

Model 4: adjusted for baseline M, place, age, education, income, and oral health behaviors

Women in intervention group had 48% [CR = 1.48 (95% CI 1.27; 1.73)] more filled teeth compared to women in control group. In adjusted models, people with low or middle SES had more D, M, and F compared to control group (P < 0.05) (Tables 5).

**Table 5:** Association between demographic and dental care behaviors with number of filled teeth at 24 months follow up (n = 436)

F-24 months		Model1 P-value and CR (95% CI)	Model2 P-value and CR (95% CI)	Model3 P-value and CR (95% CI)	Model4 P-value and CR (95% CI)
Baseline F at pregnancy		< 0.001*	< 0.001*	< 0.001*	< 0.001*
Place	Intervention	< 0.001* and 1.48 (1.26, 1.73)	< 0.001* and 1.49 (1.27, 1.73)	< 0.001* and 1.51 (1.29, 1.76)	0.03* and 1.19 (1.01, 1.41)
age			0.04*	0.78	0.60
Family size				0.20	0.41
Education				0.79 and 1.00 (0.98,1.02)	0.78 and 0.99 (0.97,1.01)
income	Low			< 0.001*	0.009*
	Middle			0.009* and 1.30 (1.06,1.59)	0.03* and 1.24 (1.01,1.52)
Daily brush (at 6 m)	Less than once				0.10 and 0.85 (0.70,1.03)
Daily floss(at6m)	Less than once				0.93 and 0.99 (0.82,1.18)
Having Visited a dentist in the past 12 months (at 6 m)	Yes				< 0.001* and 2.14 (1.81,2.53)

GLM model with Poisson link.function

Bold numbers: relationship significant at 5% level

Model 1: adjusted for baseline F and place

Model 2: adjusted for baseline F and place, and age

Model 3: adjusted for baseline F, place, age, education, and income

Model 4: adjusted for baseline F, place, age, education, income, and oral health behaviors

## Discussion

We designed this study to assess the effectiveness of some modified educational/behavioral interventions aimed at oral health promotion in pregnant women. Our particular interventions were feasible, cost effective and not complicated that made them applicable especially in deprived areas like this study’s field. Also, some interventions were done by PHCPs in order to integrating oral health into general health as the new FDI World Dental Federation’s manifest recommended [13].

In our study, the baseline mean DMFT was higher in the intervention group compared to the control group and remained higher in the last follow-up due to the nature of DMFT index; it means that DMFT is a cumulative index and never decreases over time. The DMFT index in the intervention group was higher than the control group due to the increase in M and F and decrease in D components. In other words, contrary to the control group, mothers in the intervention group followed their dental visits and let the dentists to treat their decayed teeth. Also, we observed brushing habit was significantly increased in the intervention group while it did not change in the control group at the last follow-up. This improvement in brushing behavior was also observed in other studies [21–24].

Sweet consumption behavior and having regular dental visits are usually affected by pregnancy and post-delivery period. Lactating women usually ignore their own nutrition and health after delivery because they are very busy taking care of their newborns [25]. But mothers in the intervention field had more dental visits and less sweet consumption in the last follow-up. Our result substantiated the findings from the previous studies which indicated that educational intervention may increase mother’s awareness of healthy nutrition, leading to lower sugar consumption [12, 22, 26].

In the present study, there was a significant positive association between family size and M, which has mentioned in a paper discussing World Health Organization global policy for improvement of oral health [27]. Moreover, in most low- and middle-income countries, investment in oral health care is low [28]. This association may be resulted of some caries risk factors at the population level include low family income, restricted dental care access, and low oral health knowledge that involves low-income groups [29].

We conducted four types of interventions to evaluate the effectiveness of different educational methods and messengers. D, M, and F changes were more noticeable in comprehensive intervention group compared to other intervention and control groups. The highest reduction of D and the highest amount of M, and F increase were observed in the comprehensive group compared to others. These findings are in accordance with the results of an Iranian study performed via an educational comprehensive intervention using lecture, group discussion, question and answer and educational videos which led to a decrease in the growth of the DMFT compared to the control group [30]. Women in comprehensive intervention group also had the highest frequency of brushing and flossing, dental visit and the lowest frequency of sweet consumption compared to other intervention and control groups. This finding is in accordance with the results of an Australian study which reported that mothers in combination intervention group brushed their teeth two or more times a day at a higher rate compared to control group and other intervention groups (oral health videos together with a bag containing an oral health pamphlet plus a toothbrush and toothpaste) [31]. We should suggest that the combination of using different methods and various healthcare providers and professionals for intervention, can lead to better transmission of oral health messages to mothers, ultimately leaving a cumulative effect on mother’s oral health behaviors.

Women in PHCPs intervention group had the same number of filled teeth as comprehensive group at the last follow-up. This type of intervention acts better in D reduction and M incremental changes and more frequency of brushing habit and dental visit and less sweet consumption compared to social network and group discussion led by dentist interventions. Agreed to our findings, another study conducted educational oral health interventions by midwives as PHCPs, reported significant increases in F and decreases in D were observed in intervention group compared to the other group [32]. Two other studies also reported increasing in oral health care habits of mothers who trained by prenatal healthcare providers in intervention groups compared to control group [22, 33]. Owing to mothers' frequent access to maternal and child healthcare during pregnancy and 2–4 years after delivery of their children (such as vaccination time), and also covering a large target population, especially expectant and new mothers and their children at a very low cost, PHCPs are uniquely positioned to provide oral health education, and referrals of mother for utilization of dental services and play a significant role in mothers' and children's oral health.

Group discussion performed by dentists was more effective in improving oral health care and treatment seeking behaviors and also decreasing dental caries compared to the control group and social network in this study. a study revealed that at the end of the study mothers, who received oral health education and discussion session by dental professionals, had more brushing and flossing habits compared to control group [24]. Dentists, as an oral health professional, can reliably diagnose dental caries and advising mothers to seek dental treatment. They are in an ideal position to train and motivate mothers on their oral health and assist them to carry out brushing and flossing in a proper and efficient way.

From pregnancy period up to the last follow-up, participants in social network intervention group had the least changes in D, M, and F and also oral health care behaviors. But some other studies reported significant improvements caused by social networks educational interventions. A Brazilian study reported that the use of mobile applications as a tool for delivering oral health promotion can improve oral health knowledge and decrease oral health indices [34]. Also, Scheerman et al. performed promotional oral health intervention study on adolescents and their mothers. Mothers received social media and behavioral education. After the first and the 6th month follow-up, the frequency of tooth brushing among adolescents in both intervention groups were significantly higher than the control group [35]. Based on recent studies, using mobile social network can improve people's oral health awareness and promote oral health behavioral more effectively. Nevertheless, our result showed that using social network was not as presumably effective as it was thought This may be because lactating mother are busy taking care of their new born and do not spend much time to check oral health messages popped up on their mobile phones. Despite our finding, because of cost effectiveness and availability of this method, we suggest applying it in the pregnant women’s oral health promotion efforts in this country.

## Limitations

We should mention that due to the different culture and structure of the healthcare system in the Islamic Republic of Iran, our findings might not be generalized to other countries. Nevertheless, it may be suitable to be considered for similar deprived areas in Iran.

Unfortunately, due to the limited population of Pishva and Pakdasht, we could not collect enough samples to fulfill the participants’ estimated size, so the study is underpowered (CI is broad due to this issue).

Also, it should be mentioned that for those who did not participate in the study, the Comprehensive Health Centers did not provide patient information to the study team due to confidentiality.

We should acknowledge that random assignment was not applicable in this study due to geographical proximity of residence’s location in each area that increased chance of data transmission between intervention groups (sample contamination).

Some women were not reachable for social network intervention because they did not have smart phones or did not use social applications.

## Conclusion

It can be concluded that, our interventions not only improved mothers ‘oral health behaviors such as brushing, nutrition, and dental visit in the previous 12 months, but also led to reduction of decayed teeth and filling untreated teeth in intervention group in a feasible and applicable manner and they complied with new oral health promotion suggestions made by health organizations.

Likewise, our comprehensive preventive intervention demonstrated improved maternal oral health, in a cost-saving manner. Our results suggest Performing educational interventions during and after pregnancy using both oral health professionals and trained PHCPs at the local context (Iran), could help the promotion of oral health status and awareness of pregnant and lactating mothers.

## Data Availability

The data that support the findings of this study are available from the corresponding author upon reasonable request.

## Abbreviations

CI: Confidence intervals
D: Decayed teeth
M: Missing teeth due to caries
F: Filled teeth due to caries
OR: Odds ratio
PHCPs: Primary Health Care Providers’
SES: Socio-economic status
SD: Standard deviation
WHO: World Health Organization
